# Fretting Fatigue Performance of Unidirectional, Laminated Carbon Fibre Reinforced Polymer Straps at Elevated Service Temperature

**DOI:** 10.3390/polym13193437

**Published:** 2021-10-07

**Authors:** Danijela Stankovic, Luke A. Bisby, Zafiris Triantafyllidis, Giovanni P. Terrasi

**Affiliations:** 1 Institute for Infrastructure & Environment, School of Engineering, University of Edinburgh, Mayfield Road, Edinburgh EH9 3JL, UK; Luke.Bisby@ed.ac.uk (L.A.B.); giovanni.terrasi@empa.ch (G.P.T.); 2 Empa, Swiss Federal Laboratories for Materials Science and Technology, Überland Str. 129, 8600 Dübendorf, Switzerland; Zafeirios.Triantafyllidis@empa.ch

**Keywords:** unidirectional (UD) composite straps, pin-loaded, fatigue performance, S–N curve, service temperature, carbon fibre-reinforced polymer (CFRP) straps

## Abstract

The fretting fatigue performance of laminated, unidirectional (UD), pin-loaded, carbon fibre-reinforced polymer (CFRP) straps that can be used as bridge hanger cables was investigated at a sustained service temperature of 60 °C. The aim of this paper is to elucidate the influence of the slightly elevated service temperature on the tensile fatigue performance of CFRP straps. First, steady state thermal tests at ambient temperature and at 60 °C are presented, in order to establish the behaviour of the straps at these temperatures. These results indicated that the static tensile performance of the straps is not affected by the increase in temperature. Subsequently, nine upper stress levels (USLs) between 650 and 1400 MPa were chosen in order to establish the S–N curve at 60 °C (frequency 10 Hz; R = 0.1) and a comparison with an existing S–N curve at ambient temperature was made. In general, the straps fatigue limit was slightly decreased by temperature, up to 750 MPa USL, while, for the higher USLs, the straps performed slightly better as compared with the S–N curve at ambient temperature.

## 1. Introduction

The use of carbon fibre-reinforced polymers (CFRPs) is well established in applications where high strength and rigidity, low weight, and durability are important, e.g., in the aerospace, automotive, renewable energy, and civil construction sectors [1,2]. It is, thus, not a surprise that, since their first use in the 1970s, their production growth has continued to increase, by around 12% per year, with an annual CFRP production of around 10^5^ tons per year [3]. Regarding construction applications, CFRP materials have been widely used in structural strengthening for more than two decades and are also increasingly being implemented as reinforcement or entirely composite members (i.e., girders) in new-build structural engineering applications [1,4,5]. CFRP tensile elements, in the form of unidirectional straps, have also been introduced in bridge construction; examples include a three-span footbridge in Cuenca, Spain [6], in which pin-loaded CFRP straps with stainless-steel ring terminations are used in a stressed-ribbon bridge form, and a footbridge at Empa (Dübendorf, Switzerland) [7], in which pin-loaded non-laminated CFRP straps are used to prestress a timber bridge deck in a bowstring arch typology. Further examples of structures implementing CFRP tensile elements can be found in a detailed review by Liu et al. [8].

**Figure 1:**
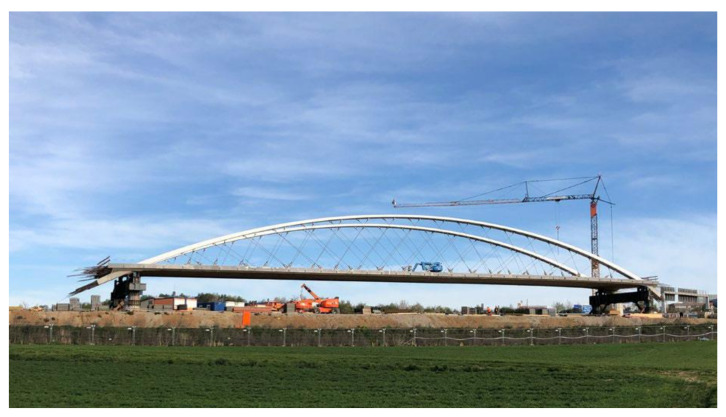
Network arch bridge with inclined CFRP cables, reprinted from ref. [9], reproduced with permission by Prof. Urs Meier.

A recent milestone regarding the application of composite elements in bridge construction is the world’s first railway (tram) bridge, in which the deck suspension relies entirely on CFRP hangers (see Figure 1); this was completed in 2020, in Stuttgart, Germany [9]. The hangers, in this case, are pin-loaded laminated CFRP straps, wound around titanium eye connectors and connected to the bridge arch in a network-tied configuration. In addition to the well-known superior non-corroding nature and durability of CFRP, Meier et al. [9] have reported that—in this arrangement—the smaller cross-section and reduced self-weight of the CFRP hangers also resulted in beneficial dynamic characteristics guarding against wind-induced vibration. They also demonstrated significant cost savings and lower CO_2_ emissions, in a life cycle assessment, when compared with conventional flat steel hangers.

Despite the fact that a small but significant number of structures relying on CFRP tensile elements have been realized to date, there remains a paucity of information on the laminated, pin-loaded CFRP straps used in the above case study, and particularly with respect to their fatigue performance when subjected to high (tensile) fatigue loads combined with a fretting process due to pin-loading at the straps’ ends. In the absence of sufficient data and design guidance, the design of such structures must be based on single-case approvals by the responsible authorities, supported by bespoke full-scale testing of the innovative CFRP elements under investigation; a description of such a procedure for the hangers of a bridge is shown in Figure 1 and given in [9].

Although CFRP materials generally exhibit comparatively good performance with respect to fatigue loading [1,10] (and, indeed, the specific hanger design in the single-case approval testing of [9] showed excellent fatigue endurance in large-scale testing), the damage mechanisms and fretting phenomena that limit the fatigue performance of pin-loaded CFRP tensile elements remain poorly understood and require further investigation. This is reflected in [9], where CFRP straps’ remnant tensile strength was reduced by up to 30% after fretting at 4.2 Hz and more than 11 million loading cycles—noting that a realistic load frequency is no more than 0.003 Hz. This corresponds to a service life of about 100 years.

### 1.1. Fretting Fatigue in UD CFRP Elements

**Figure 2:**
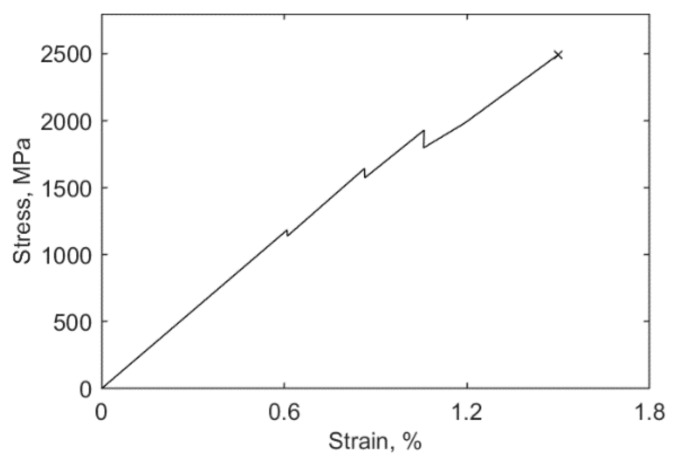
Typical stress vs. strain curve for a CFRP specimen.

Observations from static tension tests of conventional, laminated unidirectional (UD) CFRP specimens have shown that their first failure involves intralaminar crack formation in the matrix between the fibres, followed by propagation [11] starting at a so-called ‘kink’ in the strain versus strain diagram (Figure 2).

As the specimens are loaded along their fibre direction, the failure mode evolves to sudden fibre breakages. This failure mode is triggered by matrix failure and interfacial fibre–matrix debonding, parallel to the fibre direction [11]. The damage mechanisms of tension–tension fatigue tested UD specimens are similar to those observed in static tensile tests, however the final failure modes can be different, i.e., typically of a less explosive nature. Reifsnider [12] notes that fatigue damage is a cycle-dependent degradation of internal integrity, and that it can be considered as a chain of events usually of accumulative nature. These damage events include inelastic deformation (i.e., viscoelastic behaviour), microcracking and/or debonding of the matrix and its fibres (reinforcement), delamination of adjacent plies, and combinations of these events. During cyclic loading in the fibre direction, transverse cracks appear and intensify as the number of cycles increases until fibre breakage that consequently leads to fracture of the UD plies [13]. In his work, Reifsnider [13] also outlined the basic parameters that can affect the fatigue life of CFRP members, such as the mean stress *ó_m_* (or strain *ɛ_m_*) level, the stress ratio (*R*), the fibre volume fraction (*V_f_*), and frequency (*f*).

Other researchers have also identified these parameters, along with the main damage modes experienced during fatigue, namely: matrix cracking, fibre–matrix debonding, delamination, and fibre fracture [14,15]. Hahn [16] noted that cracks in tensile fatigue would naturally appear first in the plies whose fibre direction is normal to the loading direction. Hahn also stressed that matrix fatigue cracks in UD plies could grow much longer in distance and could even reach the specimens’ grip regions. Other studies also support a hypothesis that multiple-matrix cracking usually initiates at locations of defect, such as voids, areas of high fibre-volume fraction (intralaminar cracks) or resin-rich regions [17,18]. A collection of research studies on damage mechanisms in fatigue is given in [10,19]. This fundamental work distinguishes fatigue damage in three distinct phases: (1) an initial phase that corresponds to damage observed in quasi-static loading, i.e., matrix cracking; (2) a second phase, in which damage grows, interacts, and stabilizes; and (3) a third phase, in which damage combines at an accelerating rate that leads to final failure.

**Figure 3:**
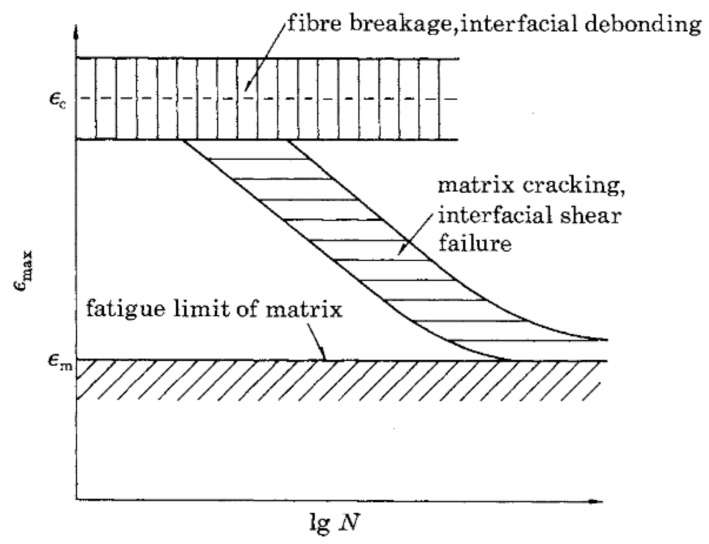
Maximum strain vs. logarithmic number of cycles to failure, reprinted with permission from ref. [20]. Copyright 1981 The Royal Society.

Talreja [20] has presented a consistent pattern of fatigue-life diagrams as an outcome of multiple and continuous observations of the damage mechanisms of UD composites. The damage mechanisms determining fatigue strength, according to Talreja [20] are shown in Figure 3 and provide a more complete understanding of the damage evolution under cycling loading, suggesting the existence of an endurance limit when the fatigue strain of the UD composite is kept below the fatigue-limit strain of the polymer matrix (being approximately 0.6% for an epoxy resin [17]). There is still debate in the composites engineering community, however, as to the existence of a distinctive edge cycle number and a respective fatigue endurance limit for UD FRP composites [21].

**Figure 4:**
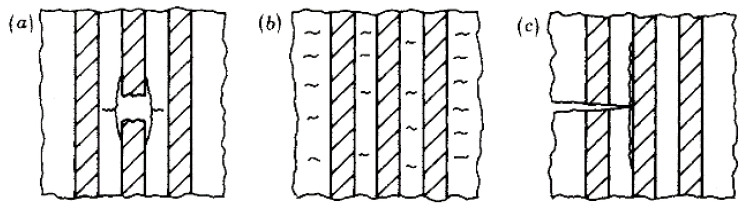
Fatigue damage mechanisms in UD composites under loading parallel to the fibre direction: (**a**) fibre breakage/interfacial debonding; (**b**) matrix cracking; (**c**) interfacial shear failure, reprinted with permission from ref. [20]. Copyright 1981 The Royal Society.

Three main regions are evident in Figure 3, each corresponding to one or more fatigue-damage mechanisms: fibre fracture/interfacial debonding (Region I), matrix cracking/interfacial shear failure (Region II), and the fatigue limit of the polymer matrix in which cracks are arrested (Region III). Each damage mechanism is schematically illustrated in Figure 4a–c. Similar fatigue failure mechanisms, and hence regions, are reported by Dharan in a study of glass fibre-reinforced composites [22].

In summary, fatigue of an FRP composite laminate, on its own, is a complex phenomenon, and, combined with fretting via pin-loading, becomes even more complex. Fretting fatigue is a surface phenomenon and the fretting process itself involves the reciprocating motion of one contacting surface (or part of one surface) over another [23]. Each fretting-fatigue problem is unique and has its own mechanics and mechanisms. A series of tests in a study by Friedrich, Schulte, and Kutter [24,25,26], investigated the fretting-fatigue phenomenon and observed how it affected the fatigue life of different types of composite specimens. Their results indicated that the fatigue life of the specimens was increased wherever 45° plies were used to protect the UD (0°) plies against fretting, and whenever the fretting-fatigue damage was concentrated towards the edge of the specimens. They also reported different damage mechanisms, under fretting fatigue, for CFRP laminates, such as wear, the thinning of the fibres, fibre fracture, and the cracking of fibre/matrix interfaces. Cirino et al. [27] later showed that the abrasive wear behaviours of polymer composite materials were affected by fibre orientation and that optimum wear resistance occurred when the fibres were oriented normally to the sliding surface.

A recent study by Baschnagel et al. investigated the impact of fretting on fatigue performance, specifically for UD CFRP-looped elements [28,29]. In their study, pin-loaded, laminated CFRP straps were tested in tensile fretting fatigue at a frequency of 10 Hz and stress ratio of 0.1, using a CFRP pin to anchor the straps. The straps used by Baschnagel et al. represented scaled-down models of the actual bridge hangers of the network arch bridge shown in Figure 1 (refer to [9]), and were compared against the tensile fatigue behaviours of two full-scale straps that were part of the structural design-type approval procedure.

SEM images of the full-scale straps revealed carbon fibre thinning and fibre–matrix debris agglomerating in the vertex area of the straps after failure [29]. Also, in [29] scans showed that the fretting products on the pin looked similar to those described in [28], in that mostly short, broken fibres and resin particles were attached to their surface; the fretting products of the model straps were mainly small carbon particles. The damage modes reported were delamination that initiated at the end of the straps’ overlap and progressed towards the curved (pin) area, followed by fibre fracture. The final failure of the straps was explosive and sudden and was initiated at the onset of the straps’ curvature through a combination of a longitudinal tensile and bending stress peaks, a compression transverse to the fibre direction, and high shear stresses [28,29]. It is worth mentioning that, for the scaled-down straps in [29], an inclusion of a sacrificial ±45° twill ply on their inner surfaces, at their curvatures (i.e., in contact with the loading pin), was also investigated. This did not, however, seem to affect the fretting-fatigue performance of the model straps or their failure modes; this being in contrast to the findings of Friedrich et al. [24,25,26] regarding the fretting behaviour of flat CFRP plate specimens with protective ±45° plies.

### 1.2. Effects of Temperature in Fatigue Performance

In fatigue tests by Baschnagel et al. [28,29], temperature development was monitored at the surface of straps in the pin-loaded region (at the vertex). Recordings of peak temperatures of up to 65 °C for the small-scale model straps, and up to 80 °C for the full-scale straps were reported. This temperature increase resulted from heat, due to friction, dissipated between the strap and the contacting pin [28,29], and molecular friction within the cyclically loaded material under high load and frequency [10,30]. Heat dissipation from fatigue loading is known to affect the endurance of FRP composites if the temperature rises near to (or indeed above) the glass transition temperature, *T_g_*, of the polymer matrix [10,31]. However, in the case of Baschnagel et al.’s CFRP straps, this was not considered critical, since the recorded temperatures were well below the glass transition temperature of the particular epoxy resin used (*T_g_* = 140 °C) [28,29]. Furthermore, these (relatively high) observed temperature rises were a consequence of the high frequencies (4.2 Hz to 10 Hz) adopted for accelerated fatigue testing in the laboratory, representing the whole lifespan of their bridge hangers [9,29]; these are not expected to be critical for the typical frequencies of real railway traffic loading, which are lower than 0.1 Hz [9]. It is worth noting that ISO 13,003 [32] recommends a maximum temperature increase of 10 °C for the fatigue testing of FRP composites.

Apart from heat building up due to the fretting fatigue phenomena at CFRP hanger connections, bridge elements in service are also subjected to temperature variations due to environmental effects, such as changes in the shade air temperature and solar radiation [33]. Therefore, a question that naturally arises is if and how the fretting fatigue performance of CFRP straps is affected at sustained service temperatures, since previous studies have only been performed at a controlled laboratory ambient temperature. Design codes [33,34] specify maximum uniform (effective) temperatures for calculating thermal actions in bridge superstructures that can be up to 16 °C higher than the maximum shade air temperature, depending on the type of construction (steel, concrete, and composite steel/concrete bridge decks). Furthermore, to account for adverse loading effects from thermal actions, Eurocode 1 specifies an additional temperature difference between the deck and suspension cables of 10 and 20 °C for light- and dark-coloured surfaces, respectively [33]. This means that, for specific climatic regions and bridge deck types, the maximum design service temperature of the hanger may exceed 70 °C.

To the best knowledge of the authors, no published data exist regarding the experienced service temperatures in CFRP bridge cables exposed to seasonal and daily temperature variations and solar irradiation (in any jurisdiction). However, significant temperature rises have been previously documented from long-term monitoring of strengthened bridge decks with externally bonded CFRP plates [35]. In a study of reinforced concrete cantilever slabs that were strengthened at their top surface with bonded CFRP strips, Czaderski et al. measured adhesive (bond line) temperatures up to 48.9 °C under direct sun exposure during hot summer days in Dübendorf, Switzerland (a difference of up to approximately 15 °C from shade air temperature) [35]. In this case, the CFRP strips were somewhat protected from UV radiation and humidity with a light-grey polyurethane coating. At the same time, Czaderski et al. measured temperatures in slabs where the CFRP strips were covered by an asphalt layer (i.e., a dark surface absorbing heat under direct sun exposure); peak temperatures measured at the surface of the asphalt were 60 °C (up to 25 °C more than the shade air temperature).

Although the exact heat transfer phenomena may be somewhat different in these cases, compared with CFRP hangers under direct sun exposure and ambient temperature fluctuations, these measurements are still indicative and highlight the importance of considering sustained service temperature in the mechanical performance of these elements. This paper aims, therefore, to provide insights into the tensile fretting-fatigue performance of laminated, pin-loaded UD CFRP straps at elevated service temperatures.

A sustained temperature of 60 °C was selected, based on the above discussion, as a realistic *average* elevated temperature for the worst-case scenario of hot summer days. This temperature was also indicated by a panel of experts for the certification type approval of CFRP strap hangers for a new network-tied arch railway bridge that is currently in the planning/tendering phase for the Oder river crossing in Küstrin, Germany [36]. The S–N curve of small-scale straps is established herein at 60 °C and is compared with the corresponding results from tests performed at ambient temperature (in a laboratory). In addition, steady state thermal tests were performed at 24 and at 60 °C, to better understand and quantify the behaviour of the straps as temperature increases. The failure modes and the variation of the straps’ cross-section are also discussed.

## 2. Material, Strap Manufacture and Experimental Set-Up

### 2.1. Materials

Titanium pins and CFRP straps were the main components used in the present study. The material of the straps, which was in the form of a continuous unidirectional carbon prepreg tape (carbon fibres: IMS60 E13 24K 830tex [37]; epoxy resin: XB 3515/Aradur^®^ 5021 [38]), and the titanium pins (Ti-6Al-4V, Grade 5 [39]) were supplied by CarboLink AG, Fehraltorf, Switzerland. Table 1 summarizes the main material properties of both components.

### 2.2. Material Characterization

In this section the characterization of the composite material of the straps is presented. The fibre (*V_f_*), resin (*V_r_*), and void (*V_v_*) contents of the UD CFRP straps were determined via sulphuric acid digestion according to Method B of BS EN 2564:2018 [40]. Three samples extracted from the straight shaft length of five different straps were tested under a fume hood. Each sample was placed separately in a beaker with concentrated sulphuric acid (50 mL/sample) and heated, using hot plates, up to 160 °C (black coloration of the sulphuric acid indicated that the resin had started to break down). Subsequently, the samples were left to cool and, once at room temperature, a hydrogen peroxide solution (30 mL/sample) was slowly added, and the samples were once again heated to 160 °C, until the solution became clear and fibres rose to the surface. The beakers were then removed from the hot plates and left to cool. Finally, the contents of each beaker were filtered through a sintered glass crucible, washed with distilled water, left to dry at 100 °C overnight in an electric oven and weighed to a 0.1mg precision scale. The density of each sample was determined according to the immersion method (Method A) of ISO 1183-1:2019 standard [41] using the Ohaus^TM^ Density Determination kit manual. All samples were weighed using an Ohaus Adventurer AX324 (Ohaus Europe GmbH, Nänikon, Switzerland) analytical balance (0.1 mg precision) in a stable lab temperature of 23 °C. The fibre, resin, and void contents, as well as the density of the samples, are given in Table 2.

An average *V_f_*, *V_r_* and *V_v_* of 67.87 ± 6.26%, 28.56 ± 8.29% and 3.57 ± 2.50% were found through the chemical digestion procedure, respectively. Similarly, the average density was estimated to be 1.55 ± 0.03 g/cm^3^. The overall results are in good agreement with the fibre volume range that the supplier provided (60–65%).

**Figure 5:**
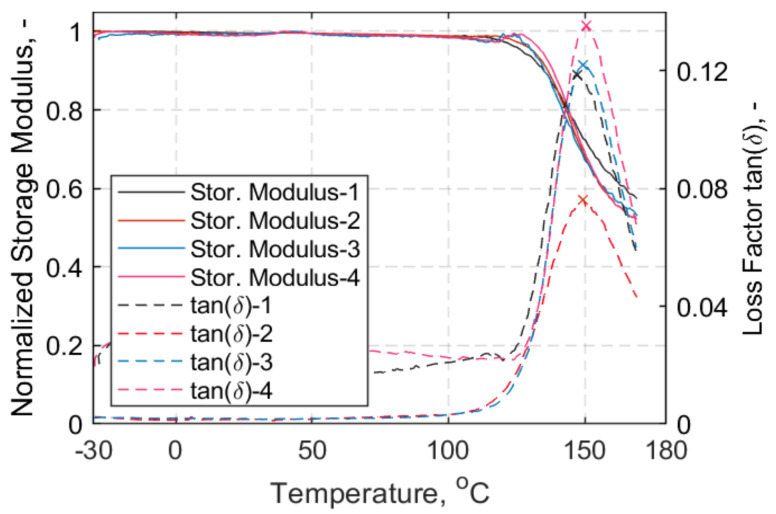
Normalized storage modulus vs. temperature (°C) (left); loss factor, tanδ (right). All samples -3PB mode.

The glass transition temperature (*T_g_*) was obtained through dynamic mechanical thermal analysis (DMTA). Four samples were analysed with DMTA using a three-point-bending (3PB) mode and imposed frequency *f* = 10 Hz with a thermal analyzer EPLEXOR 500 (Gabo Qualimeter GmbH, Ahlden/Aller, Germany). The temperature range for the DMTA tests was between –30 and 170 °C, with a heating rate of 2 °C/min. ‘Method B’, according to ISO standard 6721 [42], was followed to obtain the *T_g_* values, in this case by taking the peak value of the loss factor, tan*δ*. In Figure 5, the DMTA traces are presented and the peak value of the loss factor for each sample is indicated with an “*x*”. It is noteworthy that the *T_g_* onset, which is estimated by the intercept of the tangents below *T_g_*, appears at slightly lower temperatures, between 130 and 140 °C. The average *T_g_* value at peak tan*δ* for the four DMTA traces was 149.2 ± 1.4 °C, which is in agreement with previous measurements in [43], and within the anticipated range of 140 °C, which is suggested in the datasheet of the hot melt epoxy used herein [38]. Thus, the behaviour of the straps is expected to be rather elastic, as indicated in Figure 5, where the storage modulus and tan*δ* traces show that, at 60 °C, the viscous part of the material response is negligible.

### 2.3. Strap Manufacturing

**Figure 6:**
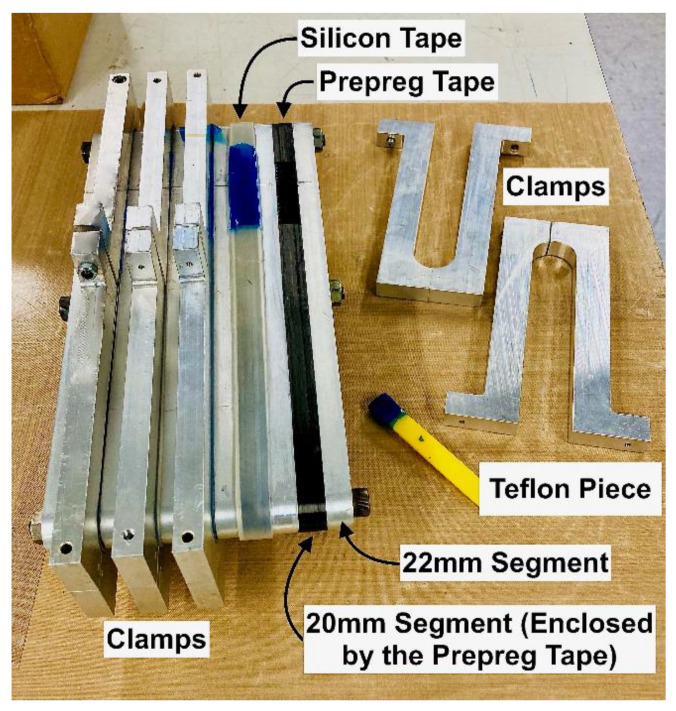
Mould, with features labelled.

The straps were manufactured by winding the continuous UD carbon prepreg tape around an aluminium mould that was then fully enclosed/compacted by aluminium clamps. At the extremities of the carbon tape an additional ±45° carbon twill ply was placed to aid in preventing delamination of the start/end points of the straps. Between the prepreg tape and the clamps a silicon tape was placed to aid in the demoulding process. The main body of the mould consisted of two types of segments: one that was 20 mm in height and enclosed by the prepreg tape, and one that was 22 mm in height, providing lateral support. The segments were placed next to each other, alternately, beginning and ending with the taller one, and three M8 bolts (fasteners) kept the assembled segments together. The assembled mould, with its main components labelled, is shown in Figure 6.

**Figure 7:**
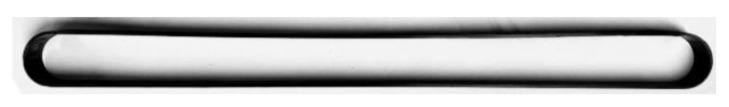
Finished strap example.

The prepreg tape was wound around the mould six times (to achieve a 1 mm thick strap) and once the winding was completed, a silicon tape was placed around it. Finally, the clamps enclosed the prepreg/silicon tape and were tightened, to provide enough pressure through the straps’ thickness during curing. The same mould geometry with a slightly different clamping system has been previously used by Baschnagel et al. [28]. Once all five straps were manufactured and clamped, the assembled mould was placed in an oven for curing. The curing cycle lasted for 3 h, since the straps first remained for 1 h at 120 °C followed by 2 h at 140 °C, following the guidance given in the manufacturer’s datasheet [38]. The bespoke titanium pins were made of Ti-6Al-4V alloy (Grade 5) and were supplied in specified dimensions. An example of a finished strap is shown in Figure 7, while the dimensions of the straps and the titanium pins used in this work are given in Table 3.

#### 2.3.1. Cross-Section Variation/Dimensional Tolerance of Straps

**Figure 8:**
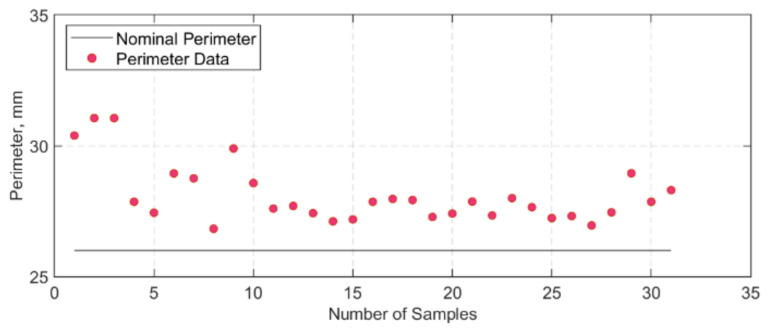
Perimeter values of each sample, after analysis in ImageJ.

**Figure 9:**
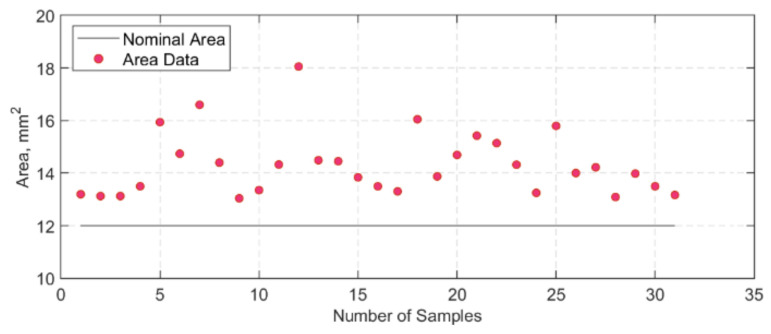
Area values of each sample, after analysis in ImageJ.

**Figure 10:**
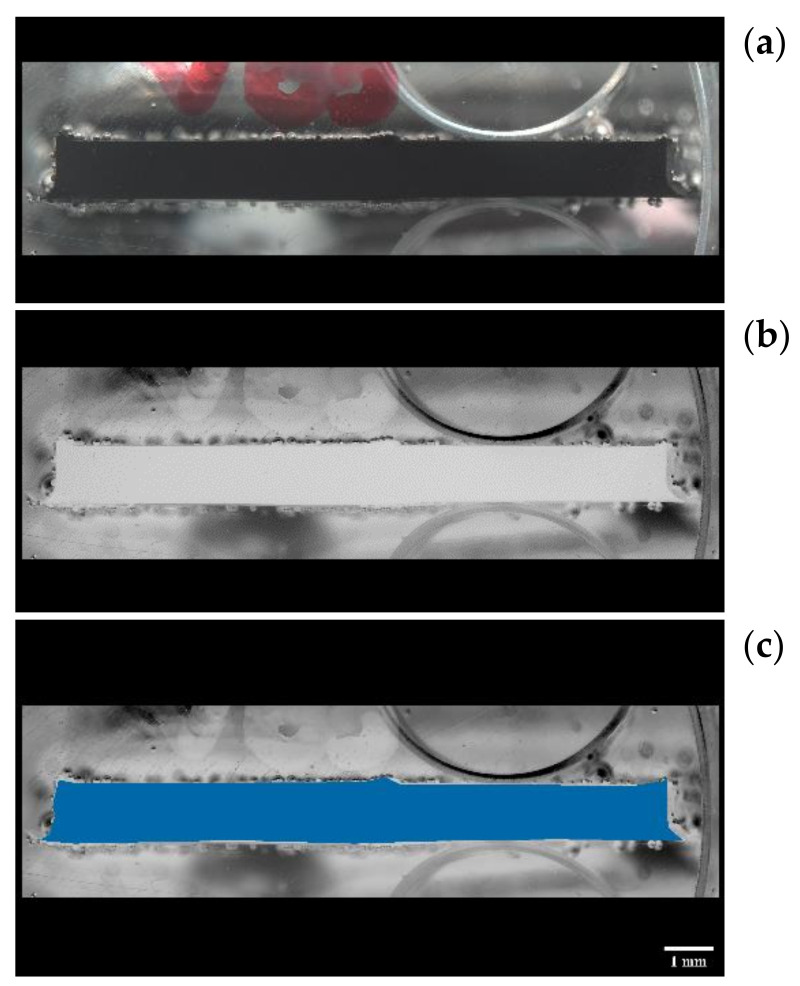
(**a**): initial image. (**b**): grayscale inverted image. (**c**): shaded RoI. Example of a cross-section of a strap used in our ImageJ analysis.

After manufacturing, the width and thickness measurements for each strap were taken prior to testing either in tension or in tension–tension fatigue. It was noted that there was some variation between the measurements, and, in some cases, there were considerable differences when compared with the nominal width and thickness (12 × 1 mm). To better capture the variation of strap cross-section, five straps were cut at the centre and at the vertex area, and six pieces per strap were examined under a microscope. In total, thirty high-resolution cross-section images of the straps were obtained, which were then processed in a Java image processing program, ImageJ [44]. The images were first converted to grayscale, and the Region of Interest (RoI) tool was used to analyse the area and perimeter chosen by the user. The resultant perimeter and area values obtained through ImageJ for each sample are presented in Figure 8 and Figure 9, respectively. The average grey threshold value used in the image analyses was 200 ± 10. An example of an image analysis is given in Figure 10.

The average area and perimeter values computed were 14.31 ± 1.21 mm^2^ and 28.10 ± 1.13 mm, respectively. When compared with the nominal (ideal) area (12 mm^2^), it is evident that the computed area from the thirty samples is approximately 15% higher. Similar observations can be made for the perimeter (nominal perimeter: 26 mm). This is significant since cross-sectional variations could affect the geometric values later used in fatigue performance calculations. For instance, the wavy and uneven perimeter of the straps suggest that during the curing cycle of the straps—where there is constant pressure from the clamps—there is material run-off from one region to another. Thus, measurements of the gross area of the straps can differ locally depending on where the micrometre is used to measure the width and/or the thickness, resulting in a variation in the estimation of the upper stress level.

### 2.4. Experimental Set-Up

**Figure 11:**
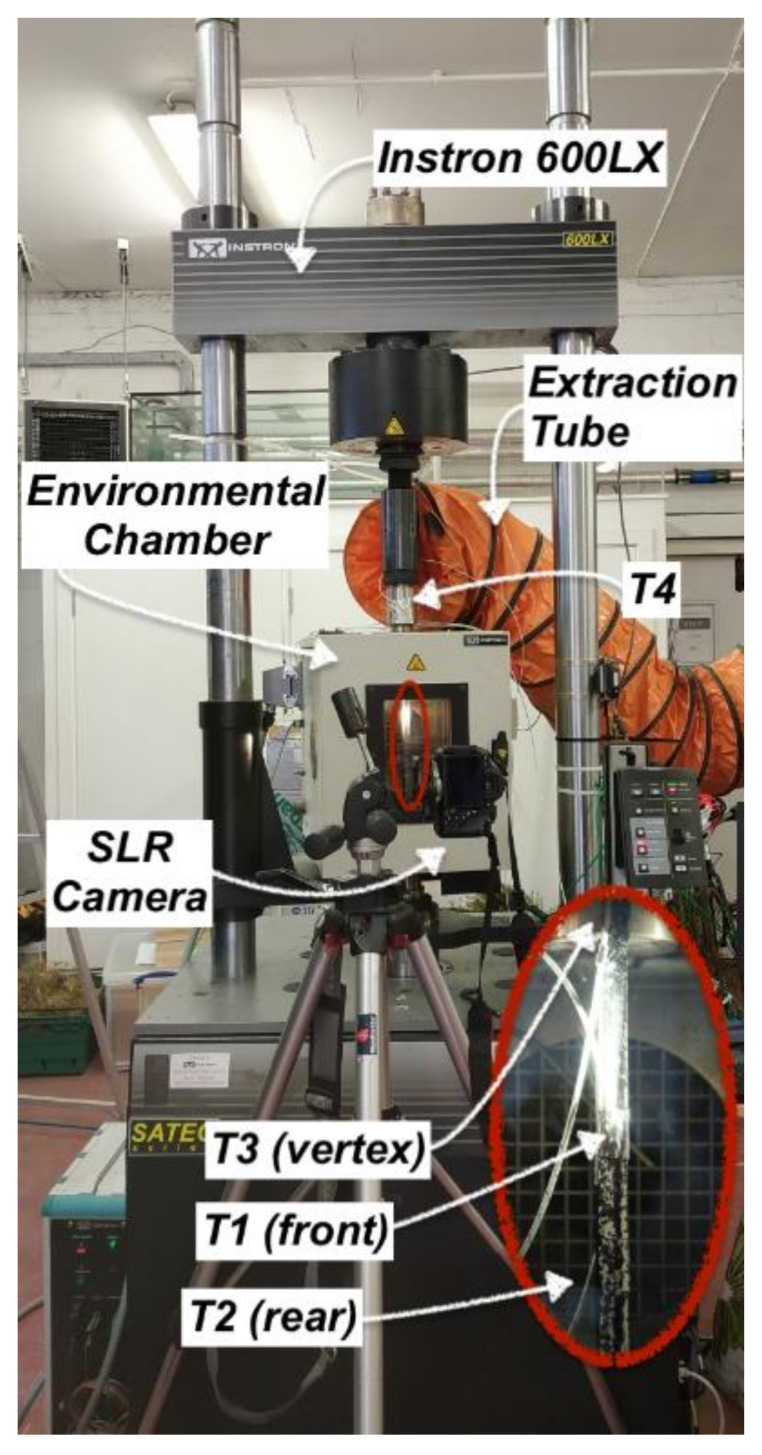
Test set-up for SS tests, with details of thermocouples’ position in the chamber (bottom right).

**Figure 12:**
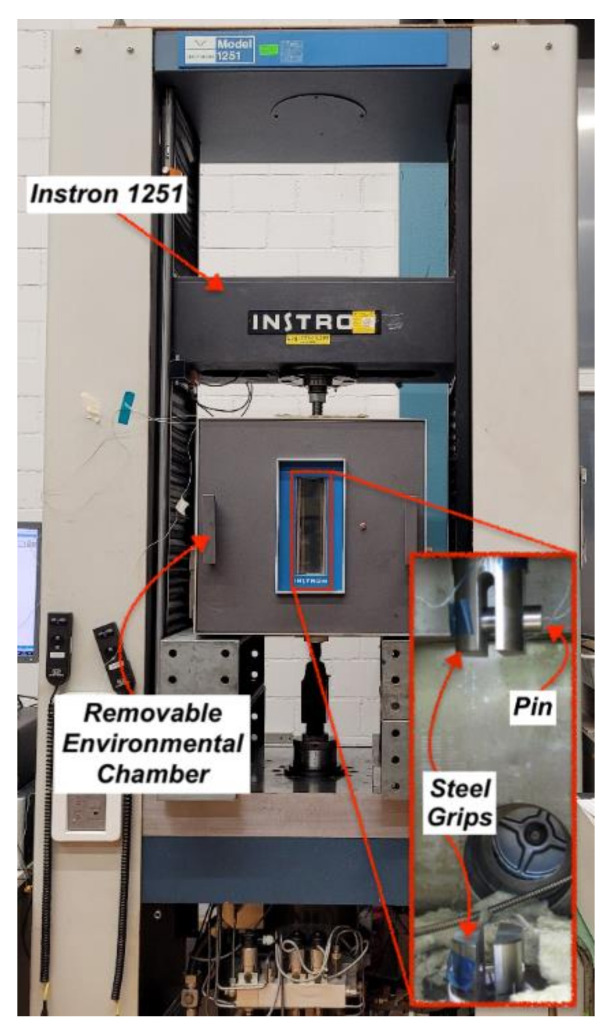
Test set-up for fatigue tests at service temperature (60 °C).

Two main set-ups, and hence two different machines, were used in this work, as the elevated temperature static tests were performed at The University of Edinburgh (UK), while the fatigue tests at sustained temperatures—and subsequent post-fatigue tensile tests—were performed at Empa, Switzerland (refer to Figure 11 and Figure 12). Regarding the elevated temperature static tests, an Instron 600LX hydraulic universal testing machine with an integrated environmental chamber was used (load capacity: 600kN, upper temperature limit of chamber: 600 °C). This is, however, not a fatigue-rated machine. During the steady state thermal (SS) tests, the strain and deformation fields (DIC RoI in Figure 11) of the straps were obtained using digital image correlation (DIC) analysis with a Canon EOS 600D camera and a remote trigger timer at a sampling rate of 0.3Hz. The DIC data were processed using Python and Matlab R2019b. Four type-K thermocouples were used to monitor the temperature development: three inside the environmental chamber and one outside. Thermocouples 1 and 2 were placed at the central region of the front and rear (closest to heating elements) shaft length of the strap, respectively, while Thermocouple 3 was located at the vertex area of the strap. Thermocouple 4 was placed on the top pull rod outside the environmental chamber. The test set-up is presented in Figure 11 and is the same as the one used in [43]. The steady state thermal (SS) experiments involved two target temperatures, namely 24 (ambient) and 60 °C. The choice of 60 °C as a target temperature was based on the sustained service temperature at which the tension–tension fatigue tests were performed. In the SS tests, the straps were loaded and kept at a constant tensile load of 0.5kN under load hold mode, until the target temperature was reached. They remained for 10 min more at the allocated temperature to ensure even temperature distribution in the strap. Subsequently, the hold mode was changed to displacement control mode and the straps were loaded until failure, with a displacement rate of 2 mm/min. Five tests per target temperature (24 and 60 °C) were performed.

The fatigue tests at elevated service temperature (60 °C) were performed using an Instron 1251 (load capacity: 250 kN) machine with a removable Instron (upper limit temperature: 300 °C) environmental chamber under load control mode, as seen in Figure 12. A frequency *f* = 10 Hz and a load ratio *R* = 0.1 were used in all tests. In addition, two type-K thermocouples were used to monitor the temperature progression: one located at the apex of the strap and one inserted in the centre of the pin, which was previously drilled (1 mm hole) to accommodate the thermocouple. In all fatigue tests, the straps were loaded on titanium pins, which were then placed in the pull rods inside the environmental chamber (refer to Figure 12). Prior to fatigue testing, the temperature inside the oven would first reach the allocated 60 °C (±2 °C) and remain additionally for 10 min to make sure the temperature across the strap was as even as possible. After 10 min had passed, the fatigue test was started. Nine different upper stress levels (USL) were chosen, namely 650, 700, 750, 800, 850, 1000, 1150, 1300 and 1400 MPa, and five tests per USL were performed in order to establish an S–N curve at 60 °C (except for 1400 MPa, at which two tests were performed instead of five). The results are compared with the S–N curve at ambient temperature previously reported by Baschnagel et al. [28]; this was obtained using a CFRP pin instead of a titanium pin.

## 3. Results and Discussion

### 3.1. Steady State Thermal Tests Results

**Figure 13:**
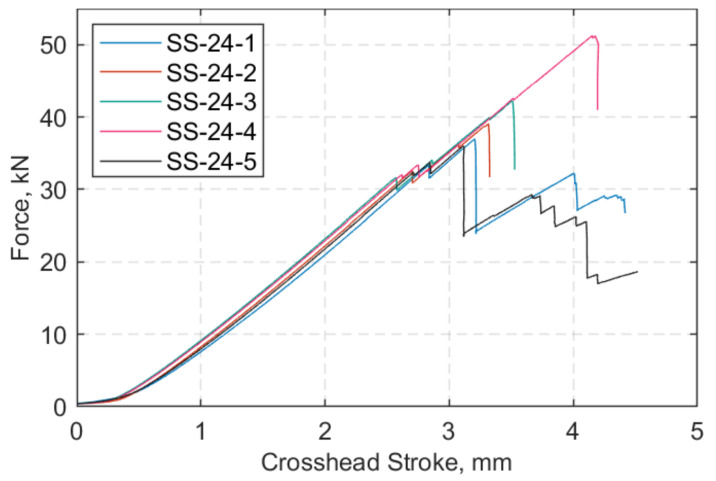
Force (kN) vs. crosshead stroke (mm) at 24 °C.

**Figure 14:**
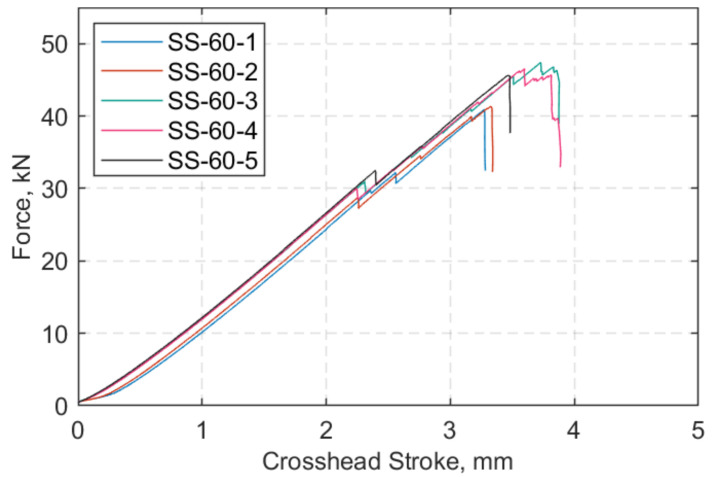
Force (kN) vs. crosshead stroke (mm) at 60 °C.

At both target temperatures of 24 and 60 °C, five tests were performed. The axial force versus crosshead displacement is shown in Figure 13 and Figure 14 at 24 °C and 60 °C, respectively. The hold mode at 0.5 kN is excluded in Figure 14. At both temperatures, there was initially delamination of the outer ply of the inner side of the straps at approximately 30–34 kN, which appears as the first ‘step’ in the figures below. The straps then, as the load was increased, exhibited increasing fibre breakages, with the final failure being sudden and explosive in nature.

**Figure 15:**
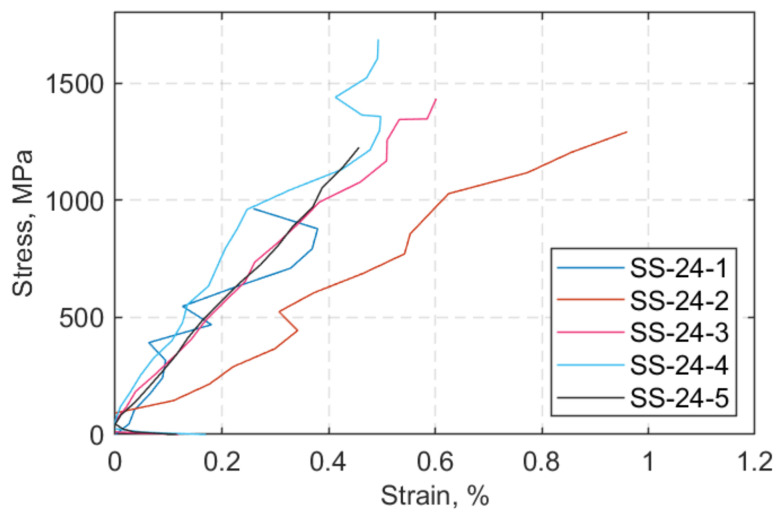
Stress (MPa) vs. strain (%) at 24 °C.

**Figure 16:**
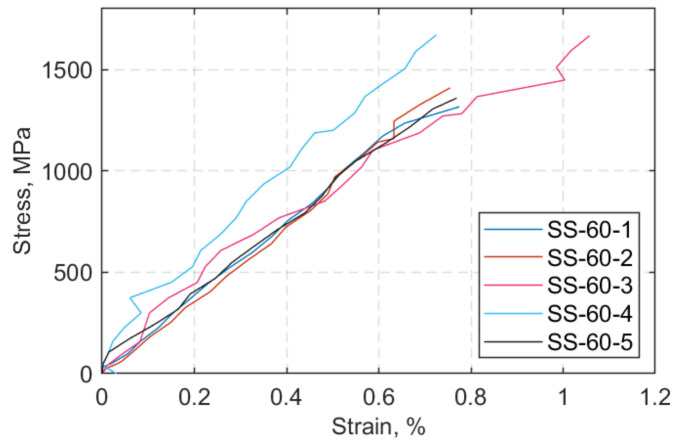
Stress (MPa) vs. strain (%) at 60 °C.

The stress-versus-strain responses of the straps at 24 °C and 60 °C are shown in Figure 15 and Figure 16, respectively. In all the SS tests, the specimen-naming notation designates SS as steady state thermal test, 24 or 60 as the target temperature, and 1… 5 indicates the number of the strap tested. The jumps that appear in some DIC strains are attributed to occasional broken fibres being present within the region of interest (indicated as DIC RoI in Figure 11) and/or out-of-plane motion—due to partial delamination of the outermost ply of the straps. Another reason might owe to the resolution of the camera (72 dpi), which, in combination with material debris in and around the region of interest (RoI). led to the “shaking” effect of the stress–strain curve.

The estimated mechanical properties of the straps tested at both target temperatures are shown in Table 4. As it can be seen in Table 4, the increase in temperature from 24 to 60 °C did not seem to have any significant impact on the static tensile performance of the straps. With respect to ultimate failure load (*F_max_*) and strength (UTS), the straps seemed to exhibit a slightly (6%) improved performance at 60 °C compared with ambient temperature. This small rise could be the result of the temperature rise, in that as the temperature increased, it allowed for stress redistribution to occur, since the matrix was more viscous due to reductions in stiffness of the epoxy with increased temperature. Such a behaviour for polymer resins—in this case, epoxy—is anticipated as, at lower temperatures, the matrix is totally brittle, while there is significant plastic deformation at 60 °C (matrix softening) [45]. The secant modulus (*E_11_*) was calculated at 0.05–0.25% strain, with the corresponding stresses calculated according to ISO 527-5 standard test method [46].

The resultant longitudinal modulus at 60 °C does not seem to be affected by the increase of temperature at the SS tests and appears to display less scatter. Based on observations from the SS tests presented in Table 4, it was expected that the straps in the fatigue tests at 60 °C should exhibit similar performance as at 24 °C, provided that no additional unforeseen damage mechanisms came into play during fatigue loading.

### 3.2. Fatigue Tests Results

**Figure 17:**
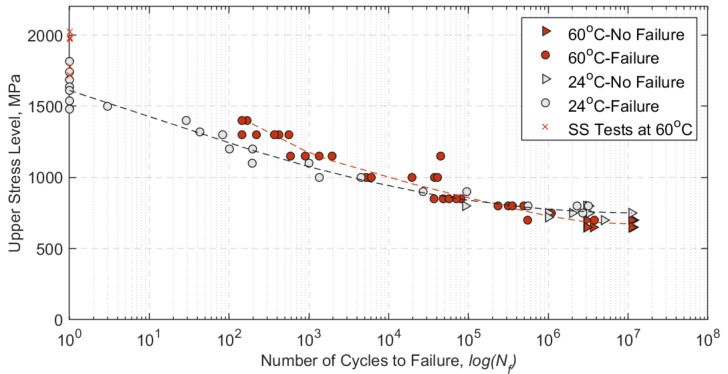
S–N curves with logarithmic best-fit (R2 = 0.93) at 60 °C and at 24 °C (reproduced with permission from [28]). UTS values from SS tests at 60 °C (N = 1) are also included.

The main objective of the fatigue tests at a sustained service temperature of 60 °C was to establish whether the straps’ fatigue life was affected by the elevated service temperature condition. This outcome is illustrated in Figure 17, where the S–N curve at 60 °C is compared with that determined for the same straps by Baschnagel and colleagues at ambient temperature [28].

The S–N curve at 60 °C shows an improvement at USLs above 800 MPa, with a small decrease of the endurance limit (of around 50 MPa) when compared with the S–N curve at 24 °C. The main difference between the strap/pin systems investigated here and in [28], besides the temperature at which the tests were performed, is the pins’ material. In the present work, titanium pins were used, while Baschnagel et al. used CFRP, pultruded pins. The choice of titanium over CFRP pins was due to their increase in service temperature, as having the adversely affects CFRP pins via extensive surface wear from the CFRP pins, which can lead to premature failure of the straps. The material removal from the CFRP pins was, at times, so intense that the pin was jammed at the grip region.

**Figure 18:**
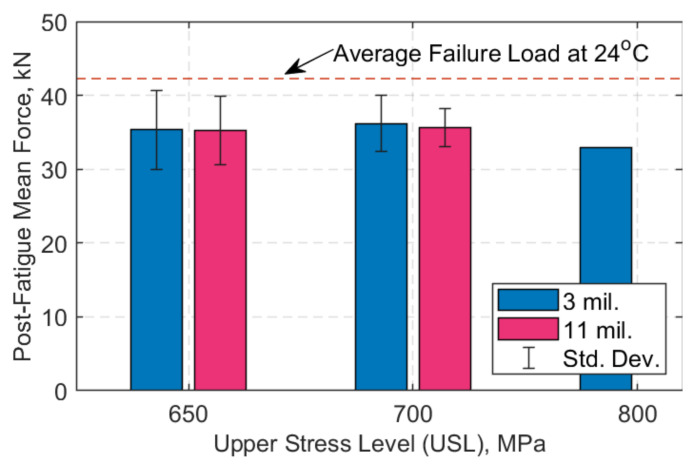
Average force (kN) vs. upper stress level (MPa); post-fatigue.

The straps that endured 3 and/or 11 million loading cycles were further tested in static tensile tests to obtain their residual post-fatigue loading strength; the results are shown in Figure 18, where the average and standard deviation values—where applicable—are also presented.

The remnant strength of the straps at ambient temperature, at an upper stress level (USL) of 650, 700 or 800 MPa for 3 and/or 11 million loading cycles, is presented in Table 5. Three straps at 700 MPa, at both 3 and 11 million loading cycles, were tensile-tested post-fatigue, while at a USL of 650 MPa, four straps were tested after 3 and 11 million loading cycles. At USL of 800 MPa, only one strap was tested to assess the remnant strength after 3 million loading cycles, since the rest of the straps failed before 3 million cycles.

The results in Table 5 show that the straps exhibited a relative decrease in their UTS—compared with the UTS at ambient temperature—after enduring either three or eleven million loading cycles at a sustained air temperature of 60 °C. The average residual strength (in percentage) compared with the ambient temperature UTS is shown in Table 6, and it is clear that about 20% of tensile strength was lost after fretting fatigue at 60 °C.

When compared with the remnant strength of the straps tested at ambient temperature in [28], at a USL of 750 MPa after 3 and 11 million loading cycles (straps B15 and R70 in [28]), it is evident that the temperature had an impact on the residual properties of the straps. However, multiple factors can affect the straps’ fatigue performance, such as the variation in the cross-sectional area of the straps. The nominal area of the straps was 12 mm^2^ and was assumed to be flat. This, however, is not the case since there is a certain waviness or uneven surface in the straps’ cross-section that when loaded on the pins might not be *fully* in contact with them. In Figure 9, there is around 15% gross area difference between the nominal area and the actual straps used in the tests.

**Figure 19:**
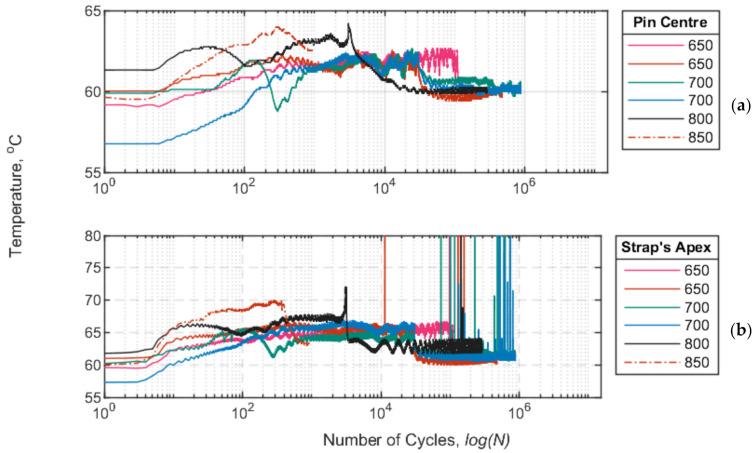
Temperature (°C) vs. number of cycles (logN) at the pin’s centre (**a**) and at the strap’s apex (**b**) for USLs between 650 and 850 MPa.

The temperature development during the fatigue tests, at a sustained temperature of 60 °C, was obtained at the straps’ apex and at the centre of the pins. More specifically, the pins were drilled at the centre of their cross section to such a length that the tip of the type-K thermocouple was located at the centre of the contact region between the pin and the strap. Representative curves of the temperature development of the two thermocouples used in the fatigue tests for USLs of 650, 700, 800 and 850 MPa are shown in Figure 19. It is evident that the constant air temperature of 60 °C during the fatigue tests did not seem to affect the titanium pins, as seen in Figure 19 (top). On the other hand, the straps seemed to experience, overall, a 5 °C gradual temperature increase at the strap’s apex during the loading cycles, which, however, did not seem to influence either their fatigue performance or their remnant strength (refer to Figure 18). The reason that the temperature profiles do not all commence at 60 °C is that there was a ±2 °C deviation in the chamber’s air temperature.

An increase in the USL (i.e., 850 MPa) does, however, affect the temperature progression of the straps, as there is a gradual and steady increase in temperature at a USL of 850 MPa. The combination of increased USL and higher recorded temperatures at the apex of the straps might have consequently led to the failure of the straps at an 850 MPa USL. This behaviour was also observed in [28], wherein a more sudden rise in temperature was noted as the USL increased from 750 to 1100 MPa.

### 3.3. Failure Modes

**Figure 20:**
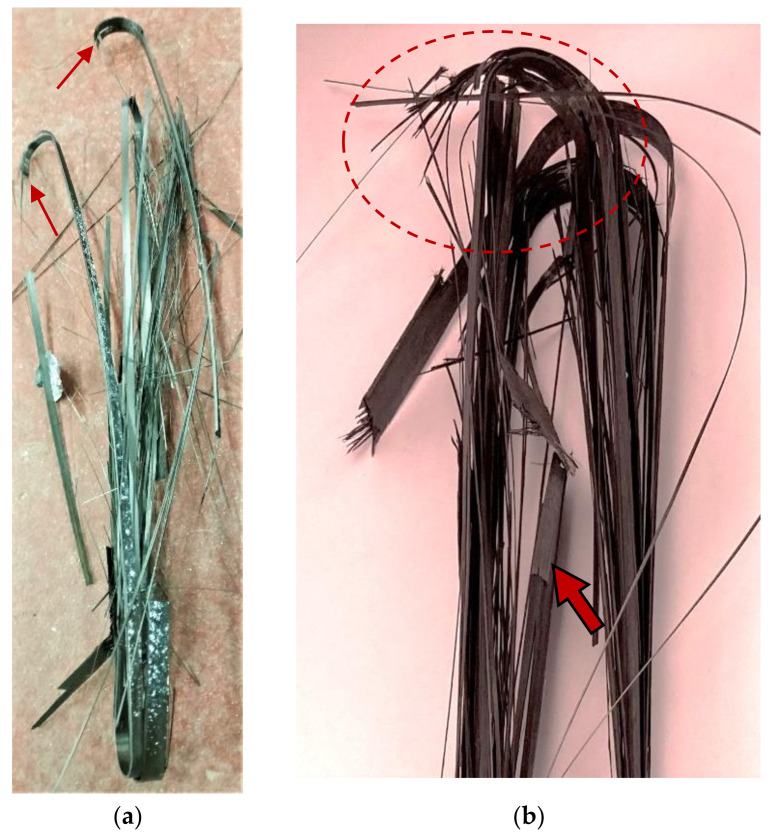
Strap failure modes in SS tests at 24 (**a**) (left) and 60 °C (**b**) (right).

The SS tests at 24 and 60 °C demonstrated similar failure modes. In both cases, initial delamination of the outer ply of the innermost layer of the straps was consistently the first visual failure mode, as indicated with an arrow in Figure 20 (right). Following, visible fibre breakages occurred and intensified as the load increased, a behaviour also reported in [28,29]. The final failure of the straps was explosive and sudden in nature, and was fibre-dominated. Such failure modes in UD CFRP specimens were previously reported by Hamada et al. [47], who also noted that the fracture process in UD specimens depends on the fibre–matrix interface and the brittleness of the epoxy resin. The initiation of the fibre breakages occurred consistently at the vertex area (stress concentration region), which is indicated in Figure 20 with arrows on the right and a dashed circle on the left and is consistent with respect to the failure mode observations made in [28].

**Figure 21:**
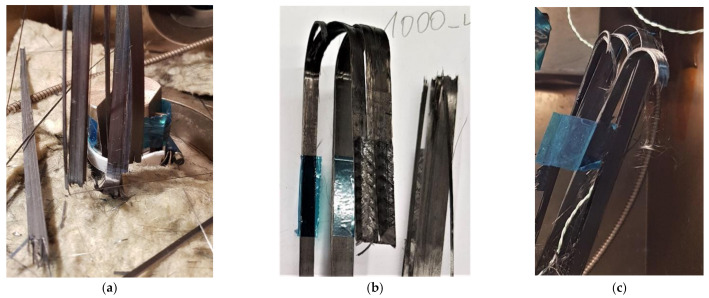
Failure mode of the straps in fatigue tests at a service temperature of 60 °C at (**a**) 1000 MPa USL, (**b**) between 1150-1300 MPa USL, and (**c**) at 1400 MPa USL.

The straps tested for fatigue exhibited similar failure modes as the straps in the SS tests as seen in Figure 21, particularly the straps at USLs of 1000 MPa and higher. At USLs between 1000 MPa and 1400 MPa, the amount of sustained tensile fatigue cycles was lower than 44,800 and a visual analysis of the contact surface of the pin to the strap showed clear signs of fretting, very similar to the ones described in [28]. This explains the observed brittle failure at lower cycle numbers for these straps under higher USLs. For the lower USLs, the influence of fretting on the amount of sustained tensile fatigue cycles was less pronounced. Ultimate failure of the straps generally occurred as clean brittle breaks (non-explosive) in the fatigue tests, as opposed to SS tests at 24 and 60 °C, where explosive failure caused individual broken fibres to be scattered around the environmental chamber.

In Figure 21, the main failure modes observed for the straps between 1000 and 1400 MPa USL were delamination and fibre breakages/splitting at and around the vertex area. There were no clear signs of the delamination propagating around the vertex area and in-between the plies, which is observed in SS tests. The failure modes experienced by the straps are in accordance with the failure modes reported by Talreja [20] that belong to Region I of the fatigue-life diagram (see Figure 3,4). In Figure 21 (middle), a longitudinal split is also evident. Similar observations, regarding the fatigue failure modes of the CFRP straps, were reported in [28].

## 4. Conclusions and Future Steps

The fatigue performance of laminated, pin-loaded CFRP straps exposed at a constant air temperature of 60 °C—chosen so as to represent credible worst-case upper service temperatures for pin-loaded straps used as hanger cables in bridge applications in Europe—was assessed in this study. Initially, the fibre volume fraction (*V_f_*) of the straps was obtained through a standard acid digestion procedure and the glass transition temperature of the straps’ material was estimated via dynamic mechanical thermal analysis (DMTA). The DMTA results confirmed that the straps’ response remained essentially elastic at 60 °C. Following, the CFRP straps were initially tensile tested at ambient (24 °C) and 60 °C steady state conditions in order to obtain their mechanical properties. The increase in temperature did not seem to particularly affect their tensile performance, as the straps exhibited slightly higher ultimate tensile strength at 60 °C. A total number of 42 straps (12 mm width, 1 mm thickness) were fatigue-tested at a load ratio *R* = 0.1 and a frequency *f* = 10 Hz up to three million loading cycles in order to establish an S–N curve at 60 °C. The upper stress levels (USL) were 650, 700, 750, 800, 850, 1000, 1150, 1300 and 1400 MPa, and, at the lower stress levels (650 and 700 MPa), fatigue tests up to 11 million cycles were additionally performed. The main conclusions that can be drawn from this study are:

▪ The static tensile performance of the straps was not affected by an increase in temperature from ambience to 60 °C.
▪ The initial failure mode observed during the fatigue tests (for the straps that failed) was initial delamination of the outer ply of the innermost layer of the strap, and ultimate failure around the vertex area; it was not explosive in nature, however.
▪ The fatigue life of the straps was not affected by temperature at higher upper stress levels, although it exhibited a slight decrease in the regime of 650–750 MPa when compared with the S–N curve derived from tests at ambient temperature.

The practical significance of the fatigue test results is very positive for the potential use of CFRP straps as bridge cables or as reinforcement components at slightly elevated service temperatures or in warm climates.

Future work should involve fatigue tests on full-scale straps at 60 °C and a comparison of the results to the model straps presented herein. This comparison could also be useful to observe whether there are differences in the results between the small and full scales. In addition, further work should investigate the effects of thermal cycling on the fatigue life of the straps. This is important, especially if the straps are intended to be used as bridge hanger cables, where the seasonal temperature fluctuations and/or instantaneous drops (e.g., sudden shading) can induce thermal stresses.

## Figures and Tables

**Table 1:** Material properties of the UD prepreg tape and the titanium pins.

IMS60 E13 24K 830tex [37]	Density (g/cm^3^): 1.79 tensile strength (MPa): 5600 Young’s modulus (GPa): 290
Epoxy Resin XB 3515/Aradur^®^ 5021 [38]	density (g/cm^3^): 1.17 tensile strength (MPa): 60 ± 1.43 Young’s modulus (GPa): 2.62 ± 0.033
Titanium Ti-6Al-4V (Grade 5), (STA) [39]	density (g/cm^3^): 4.43 tensile strength -Yield (MPa): 1790 Young’s modulus (GPa): 114

**Table 2:** Fibre, resin and void volume contents, and density of the composite samples obtained through the chemical digestion process.

Sample	*ρ_c_* (g/cm^3^)	*V_f_* (%)	*V_r_* (%)	*V_v_* (%)
1	1.562	64.99	34.08	0.94
2	1.566	75.05	19.03	5.92
3	1.519	63.57	32.57	3.86
Average	1.55	67.87	28.56	3.57
St. Deviation	±0.03	±6.26	±8.29	±2.50

**Table 3:** Pin and strap dimensions (mm).

*Titanium Pin*	
Length (mm)	63 ± 1
Diameter (mm)	20 ± 0.1
*CFRP Strap*	
Shaft Length (mm) Inner Radius (mm)	250 10
Width (mm)	12 ± 0.3
Thickness (mm)	1 ± 0.2

**Table 4:** Estimated values of maximum force (F_max_), ultimate tensile strength (UTS) and longitudinal modulus (E_11_) for the SS tests at 24 and 60 °C.

Temp.	Property	Test-1	Test-2	Test-3	Test-4	Test-5	Average	St. Dev.
24 °C	F_max_ (kN)	36.98	39.08	42.21	51.23	35.97	41.09	± 6.15
	UTS (MPa)	1646.28	1726.35	1871.70	2191.81	1527.81	1792.79	± 255.70
	E_11_ (GPa)	203.44	122.31	236.92	241.06	215.26	203.80	± 48.10
60 °C	F_max_ (kN)	40.90	41.35	47.40	46.52	45.62	44.36	± 3.02
	UTS (MPa)	1718.14	1778.53	2023.52	1980.85	1974.18	1895.05	± 136.94
	E_11_ (GPa)	199.23	176.52	234.91	225.04	160.09	199.16	± 28.24

**Table 5:** Ultimate tensile strength (UTS in MPa) with average and standard deviation values after fatigue at 60 °C for USLs 650, 700 and 800 MPa.

	USL: 650 MPa		USL: 700Mpa		USL: 800 MPa
	**3 mil.**	11 mil.	3 mil.	11 mil.	3 mil.
	1489.8	1392.9	1705.2	1524.6	1565.4
	1577.1	1580.8	1715.4	1424.8	
	1519.3	1672.6	1408.4	1672.9	
	1950.9	1719.5			
Average	1634.3	1591.3	1609.6	1540.8	-
St. Dev.	±214.2	±144.3	±174.3	±124.9	-

**Table 6:** Average remnant strength of the straps at 650, 700 and 800 MPa USL.

USL (MPa)	N (Million)	UTS_Remnant_ (%)
650	3	83.60 ± 12.60
	11	83.35 ± 10.96
700	3	85.60 ± 8.99
	11	84.21 ± 6.15
800	3	77.96

## Data Availability

The majority of the data can be found in the PhD thesis to be submitted this year to the University of Edinburgh.
